# IFN-**γ** and YAP lead epithelial cells astray after severe respiratory infection

**DOI:** 10.1172/JCI185072

**Published:** 2024-10-01

**Authors:** Bradley E. Hiller, Joseph P. Mizgerd

**Affiliations:** 1 Pulmonary Center,; 2 Department of Medicine,; 3 Department of Virology, Immunology, and Microbiology, and; 4 Department of Biochemistry and Cell Biology, Boston University Chobanian & Avedisian School of Medicine, Boston, Massachusetts, USA.

## Abstract

Ineffective recovery from pneumonia can lead to interstitial lung disease characterized by aberrant epithelial cells in fibrotic regions. In this issue of the *JCI*, Lin et al. define molecular pathways leading to the development and persistence of keratin 5^+^ (Krt5^+^) epithelial cells in the alveolar parenchyma when mice struggle to recover from influenza infection. The receptor for IFN-γ on lung epithelium was essential for the formation of aberrant Krt5^+^ cells and fibrotic lung disease. The transcription factor Yes-associated protein 1 (YAP) was necessary for persistence of these Krt5^+^ cells, and IFN-γ activated YAP in lung epithelial cells via JAK, focal adhesion kinase (FAK), and Src kinases. These findings establish a targetable pathway underlying some of the pulmonary postacute sequelae of pneumonia.

## Postacute sequelae of pneumonia

The lungs have remarkable healing abilities. Most of the time, the lungs recover from even severe infection to become histologically normal but immunologically improved, with resident memory lymphocytes and trained innate immunity that together enhance defense against future infections (1). However, a fraction of patients with pneumonia experience postacute sequelae that may include pathophysiology of the pulmonary system (with interstitial lung disease especially common) or other extrapulmonary systems (such as cognitive impairment, cardiovascular disease, and more). These complications captured the public’s attention as “long COVID” (2), but they are not specific to this type of pneumonia and were already recognized as a research priority before that recent coronavirus emerged (3). Deeper understanding of the biology underlying postacute pulmonary sequelae of pneumonia is needed (4) so that pathophysiology after acute infections can be prevented, slowed, or reversed.

Interstitial lung disease is characterized by aberrant epithelial cells and thickened fibrotic tissue. A variety of unusual epithelial cells are present, expressing incongruous markers suggesting mixed and indeterminate cell types that are inappropriately positioned, including (but not limited to) epithelial cells expressing the basal cell marker keratin 5 (Krt5) in the distal parenchyma where basal cells do not belong (5–7). Aberrant transitional epithelial cells including distal Krt5^+^ cells are found in lungs of patients with postacute fibrosis resulting from COVID pneumonia (8, 9).

The mouse-adapted PR8 strain of influenza A virus (IAV) reproducibly causes very severe infection and prolonged damage to mouse lungs involving chronic inflammation, tertiary lymphoid structures, and persistent Krt5^+^ epithelial cells in the parenchyma. A study in this issue of the *JCI* (10) reveals that the aberrant Krt5^+^ epithelial cells in mouse lungs that struggle to recover from IAV infection depend on IFN-γ and yes-associated protein 1 (YAP) signaling in the epithelium.

## Curtailing aberrant Krt5^+^ epithelium after IAV infection

In this issue of the *JCI*, Lin et al. (10) made the expected observation of ineffective lung recovery after IAV infection in mice. The effects involved substantial Krt5^+^ pod formation and goblet cell hyperplasia in the alveolar parenchyma in addition to infiltrating CD8^+^ T cells. Because CD8^+^ T cells make abundant IFN-γ during IAV infection, the authors hypothesized that IFN-γ signaling might be involved in formation of the aberrant Krt5^+^ epithelial cells. Using gene-targeting tools to ablate the IFN-γ receptor IFNGR1 in all lung epithelial cells or in Sox2-expressing airway epithelial progenitor cells (11), the investigators demonstrated that IFN-γ signaling to epithelial cells (and particularly airway progenitor cells) was essential for the formation of dysplastic Krt5^+^ cells after IAV infection. The absence of IFNGR1 on epithelial cells also improved lung recovery following IAV infection, with histologic evidence showing less fibrosis and pulmonary measurements indicating improved function. Thus, in this model of IAV infection, many of the postacute sequelae require IFN-γ signaling to epithelial progenitor cells.

Downstream of IFN-γ binding its cognate receptor, the expression of hundreds of IFN-stimulated genes is mediated by STAT1 transcription factor after its activation by JAK1/2. Lin et al. (10) found that JAK1/2 antagonists inhibited the formation of Krt5^+^ cells stimulated by IFN-γ in vitro or by IAV in vivo, suggesting essential roles for these tyrosine kinases. However, epithelial deletion of STAT1 (via Shh-cre) did not affect Krt5^+^ cells, implicating alternative transcription factors. Single-cell RNA sequencing after IAV infection revealed that Krt5^+^ cells were enriched for focal adhesion pathways (involving focal adhesion kinase [FAK] and c-Src kinase) and Hippo pathways (involving YAP and transcriptional coactivator with a PDZ-binding domain [TAZ] transcription factors). The Krt5^+^ pod cells in the alveolar space after IAV infection had phosphorylated Src (p-Src) and nuclear YAP, meaning these pathways were active there. FAK/Src signaling and IFN-γ can each activate YAP (12, 13). A tamoxifen-activated Krt5-creERT2 that targets YAP in all cells expressing Krt5 revealed that YAP was essential for the maintenance of the Krt5^+^ pods that had formed in IAV-recovered lungs. Thus, YAP was necessary for these dysplastic epithelial cells, which also needed IFNGR1 but not STAT1.

**Figure 1:**
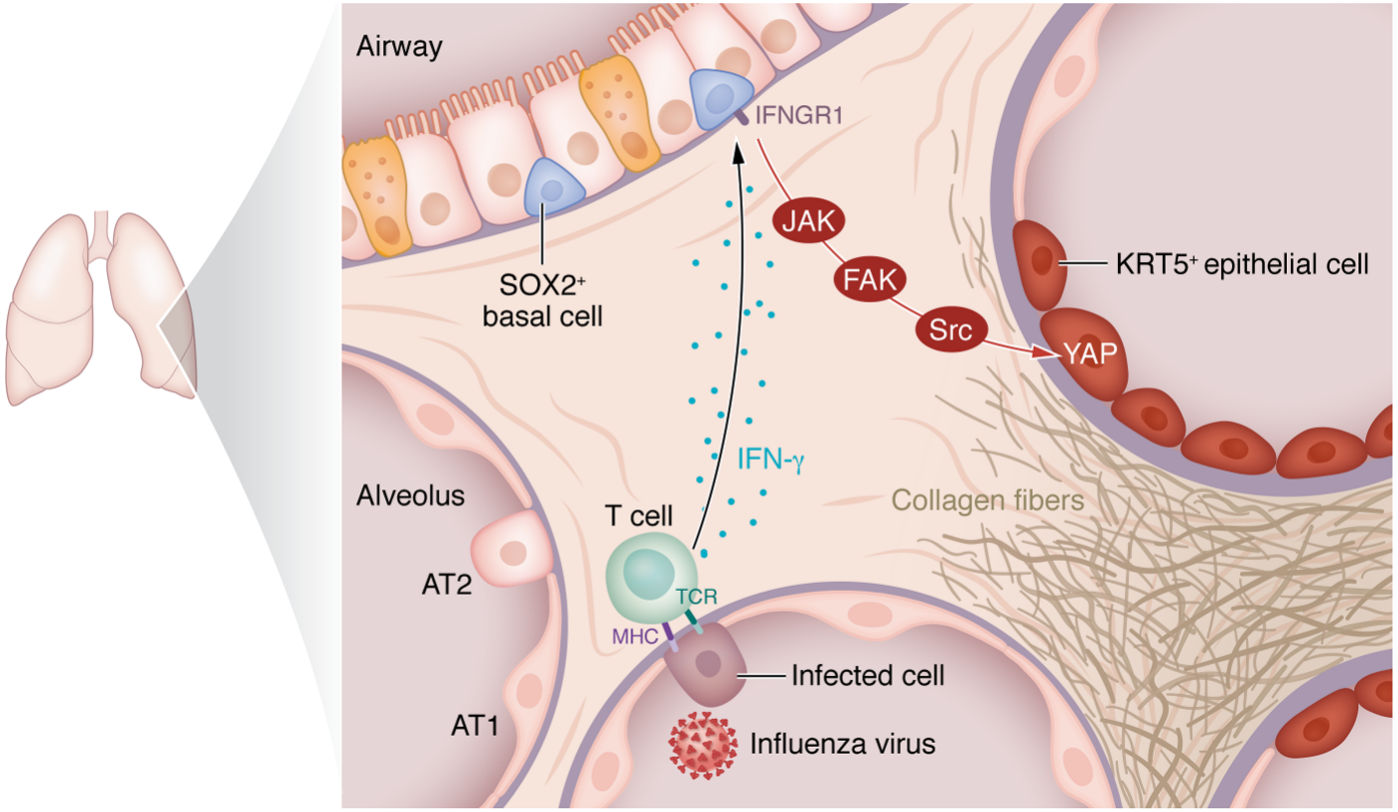
IFN-γ signaling drives dysplastic Krt5^+^ cells after lung infection. Lin et al. (10) revealed that SOX2^+^ airway progenitor cells require IFNGR1 expression following influenza A virus infection for the development of aberrant KRT5^+^ epithelial cells within the alveolar parenchyma and subsequent fibrotic lung disease. IFN-γ activates the YAP transcription factor in lung epithelial cells via JAK, FAK, and Src kinases. YAP in dysplastic, parenchymal KRT5^+^ epithelial cells is essential for their expansion and maintenance. TCR, T cell receptor.

The Lin et al. (10) findings suggest a pathway by which IFN-γ may act through JAK kinases to activate FAK/Src and then YAP to drive the formation of dysplastic Krt5^+^ cells after lung infection. Treating cultured pulmonary mouse basal cells with IFN-γ increased p-Src and nuclear YAP, which was inhibited by a JAK antagonist. Pharmacological inhibitors of FAK and Src diminished the IFN-γ–mediated YAP nuclear translocation and Krt5^+^ differentiation in these cells and also reduced the number of Krt5^+^ pods in lungs of IAV-infected mice. In human lung samples infected with SARS-CoV-2, Lin and authors observed p-Src and nuclear YAP near CD8^+^ cells. There was also transcriptomic evidence of IFN-γ receptor and focal adhesion pathway activity in aberrant transitional epithelial cells. Furthermore, these signaling interactions led to dysplastic cell formation in human lung epithelial organoid cultures. Altogether, Lin et al. have defined a mechanism that takes place in mouse or human lung during recovery from acute viral infection by which IFN-γ signaling stimulates epithelial cell differentiation into dysplastic Krt5^+^ epithelial cell pods in the alveolar space (Figure 1) along with alveolar goblet cell hyperplasia and fibrosis.

## IFN-γ and YAP mediate pulmonary responses to infection

IFN-γ is a pleiotropic cytokine made by many cells, including T lymphocytes. IFN-γ is best known for its ability to stimulate phagocyte killing of intracellular microbes. In addition, IFN-γ instructs cells to take on stable new phenotypes. For example, during acute respiratory infection, IFN-γ signaling to alveolar macrophages induces a new trained immunity state that persists indefinitely and improves lung immune defense against diverse microbes (14). Lin et al. (10) show that, after IAV infection, IFN-γ signaling to lung epithelium leads to a dysfunctional phenotype (dysplastic distal Krt5^+^ epithelial cells) as well as histopathological, biochemical, and physiological signs of lung fibrosis (10). Therefore, IFN-γ signaling to lung epithelial cells is highly pathogenic in this setting. Other recent reports identify prolonged IFN-γ release (15) and elevated IFN-γ signaling in the lung (16) as hallmarks of patients with long COVID. Blocking IFN-γ diminishes chronic inflammation and fibrosis in mice struggling to recover from SARS-CoV-2 infection (16). Altogether, converging lines of evidence suggest that IFN-γ seems to mediate at least some postacute sequelae of pneumonia. IFN-γ induction occurs during a wide variety of lung infections, most of which do not reliably cause postacute sequelae. It will be important for future studies to identify the factors that determine whether IFN-γ signaling to lung epithelial cells is detrimental or not. The amount and/or duration of IFN-γ may be key. In addition, IFN-γ signaling pathways may interact with other signaling pathways induced by infections to yield pathologic outcomes. Other signals involved in IAV-induced parenchymal Krt5^+^ cells include hypoxia-inducible factor 1α (HIF-1α) and Notch (17).

YAP and TAZ are important modulators of lung epithelial biology. Aberrant epithelial cells in fibrotic lungs are characterized by high levels of YAP/TAZ signaling (18, 19). Increasing YAP/TAZ activity, by deleting Hippo kinases in alveolar type 2 (AT2) cells (20) or by overexpressing a constitutively active form of YAP in AT2 cells (21), is sufficient to form aberrant epithelial cells in the mouse lung. Therefore, excessive YAP/TAZ in alveolar epithelial cells is harmful. However, after injury (from pneumococcal infection, bacterial lipopolysaccharide, or bleomycin), the combined absence of YAP and TAZ in AT2 cells consistently exaggerates inflammation and slows repair, worsening pathophysiology (22–24). Thus, YAP and/or TAZ in AT2 cells facilitates healthy responses to injury. In the bleomycin injury model, AT2 cell deletion of TAZ phenocopies the deletion of YAP and TAZ together; however, AT2 cell deletion of YAP has the opposite effect, diminishing inflammation and fibrosis (24). Considering that interruption of epithelial YAP while TAZ is maintained diminishes pulmonary pathophysiology after bleomycin injury (24) and curbs Krt5^+^ epithelial cells after IAV infection (10), it seems that YAP in lung epithelial cells has roles distinct from TAZ that are pathogenic in settings of injury. Future studies should determine the mechanisms by which epithelial YAP drives unhealthy outcomes including the emergence of distal Krt5^+^ cells.

The study by Lin et al. (10) provides insights into the development of aberrant epithelial cells that associate with and may contribute to lung disease, particularly postacute fibrotic sequelae of pneumonia. These findings suggest that disrupting signaling by IFN-γ, YAP, or the kinases connecting the IFN-γ receptor to YAP could potentially prove beneficial for some subsets of patients with or at risk of postacute sequelae of pneumonia. However, IFN-γ and YAP have important roles in immune defense and epithelial biology, and more precise understanding of which individuals have excessive or inappropriate IFN-γ or YAP signaling would be needed before such experimental approaches can be rationally considered.

## Version 1 10/01/2024

Electronic publication
